# Cosmic coding and transfer for ultra high security near-field communications

**DOI:** 10.1016/j.isci.2022.105897

**Published:** 2023-01-04

**Authors:** Hiroyuki K.M. Tanaka

**Affiliations:** 1 University of Tokyo, Tokyo, Japan; 2 International Virtual Muography Institute (VMI), Global, Tokyo, Japan

**Keywords:** Interdisciplinary physics, Physics methods, Properties of specific particles

## Abstract

By using true random number (TRN) generators, encoding with the highest security can be realized. However, a completely secure strategy to transfer these TRNs has not yet been devised. Quantum key distribution (QKD) has attempted to establish secure key distribution methodology of this kind; however, several quantum cracking strategies have been predicted and experimentally demonstrated. In this work, COSMOCAT was invented as a solution for next-generation ultrahigh security near-field communications. With COSMOCAT, TRNs are generated from naturally occurring and ubiquitous cosmic-ray muons and the generated cosmic keys are distributed by these muons with an unprecedented level of security. The successful results of this experiment indicate that our prototype and the new key-generation-and-distribution standard can be utilized for practical encoding and near-field data transfer at rates of 10–100 Mbps. It is anticipated that COSMOCAT will be one of key techniques for future high security, near-field communication management.

## Highlights

• COSMOCAT generates and distributes encryption keys to make the NFC more secure
• COSMOCAT generates true random number-based encryption keys
• COSMOCAT does not require information traffic for key distribution
• COSMOCAT encryption keys are not broken by large quantum computers

## Graphical abstract

**Figure:**
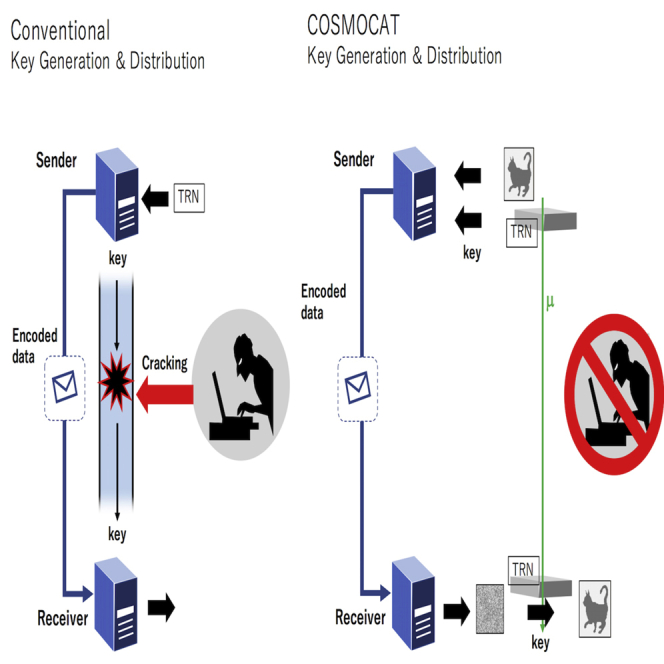

## Introduction

In recent years, near-field communication (NFC) market growth has been escalating. For example, the emergence of wireless power transfer (WPT) technology is significantly changing the traditional use of electricity.^1^ WPT is made possible by taking advantage of electromagnetic-field coupling such as inductive power.^2^^,^^3^^,^^4^ The application range of WPT is wide ranging from power stations for electric vehicles and e-scooters to operations of industrial robots. With the potential of WPT being so promising, there has accordingly been an urgent need to come up with solutions to improve security vulnerabilities of NFC networks.^5^^,^^6^

Another major concern of NFC security is associated with building automation systems (BAS). Technological advances along with a growing interest in reducing global energy consumption have boosted implementation and development of smart systems in buildings and factories. Accordingly, as urban “smartization” expands, short-range wireless communications for sensory operations will be increasingly in demand. Managing wireless NFC in smart buildings has been one of the most pronounced examples of an effective strategy to reduce the total energy consumption within buildings. Avoiding the squandering of energy in the commercial and residential sector has been a major priority recently since this sector represents ∼20%^7^ of total global energy consumption. Furthermore, with the rapid increase in population as well as economic growth, energy consumption in buildings has been increasing and is expected to continue to increase at a rate of 1.3% per year from 2018 to 2050.^8^ In response to these issues, BAS including electric lights, air conditioning systems, elevators and escalators, water pumps, boilers, security surveillance systems, etc. are installed to buildings and factories with smart technologies that can help reduce electricity by 30%–80%.^9^ Most BAS security systems are currently based on expensive and complex solutions with wired network systems. These systems are protected by relatively high security to provide real-time control and support. New solutions are required to protect the integrity of BAS.

The use of wireless technologies offers distinctive advantages for BAS. There are a number of wireless schemes designed for office networking and short-range cable emulation. Examples include Wireless LAN (IEEE 802.11, designed for the former) and Bluetooth (IEEE 802.15.1, for the latter) as well as short-range sensor/actuator networks such as Z-Wave,^10^ NanoNET,^11^ IEEE 802.15.4,^12^ ZigBee,^13^ etc. However, wireless networks are usually more exposed to security threats due to the vulnerability of the transmission medium. Wireless networks are more susceptible to security attacks than wired networks. For instance, the current Bluetooth specification defines security at the link level only, leaving the burden of selecting the most appropriate security mechanisms to each application developer.^14^ In general, the security level provided by the Bluetooth standard is too weak for it to be suitable for applications that transfer sensitive information. Regarding the aforementioned short-range wireless schemes for sensor/actuator networks, there are no security services for these protocols except for NanoNET. NanoNET offers 128-bit encryption using an undisclosed stream cipher with the support of one-time pads and message authentication. Regarding these sensor/actuator networks, there are two major security problems. First, signal integrity is not protected. Interference can disconnect the entire network with a radio frequency (RF) device in close proximity. The second challenge to improving security is related to the authentication problem. Since the RF signals are usually hard to confine, unauthorized users could gain access the network where they would then be free to exploit the connected resources.

Conventional data transfer techniques already integrate some security measures against cyberattacks.^15^^,^^16^ Quantum key distribution (QKD) is a solution to provide high security for optical communication networks which are expected to be adopted in the future. QKD is based on Heisenberg’s uncertainty principle and the quantum no-cloning theorem.^17^^,^^18^^,^^19^ QKD is believed to enable highly secured data transfer. Most of the QKD protocols employ single-photon sources and detectors for secret key generation and detection. However, since single-photon sources and detectors are still under development, implementation of QKD has been widely tested using weak and coherent light sources. Such devices are imperfect for the implementation of QKD and may cause security loopholes in the system, hence making the QKD system potentially insecure.^20^^,^^21^^,^^22^ Thus, to protect the QKD systems from such imperfections, new QKD protocols (namely, the decoy-state QKD protocol^23^^,^^24^^,^^25^ and the measurement-device-independent QKD protocol)^26^^,^^27^^,^^28^ have been proposed. The measurement device independent, for instance, can remove all of the detector-based side-channel attacks but still remains vulnerable to source-based attacks.^29^^,^^30^^,^^31^^,^^32^

The Cosmic Coding & Transfer (COSMOCAT) technique is a solution that has been developed to provide a much higher level of security for future NFC networks with simple and reliable devices. With COSMOCAT, cosmic-ray muons deliver true random numbers from the sender to the receiver. There is no physical exchange of information between the sender and the receiver. Moreover, the feasibility of utilizing cosmic rays to create cryptographic keys has already been proven and established by a number of prior works.^33^^,^^34^^,^^35^ It is generally assumed that galactic cosmic rays have a random arrival time distribution. Therefore, resultant secondaries would also have a random arrival time distribution. Cosmic-ray precipitation follows the Poissonian process. The Poissonian process is defined as one event that occurs completely independent from the occurrence of another event. Ahlen et al.^35^ evaluated 407,420 high-energy muon arrival times, and found that there were no indications of deviations or time anisotropies in nanosecond to second timescales. Therefore, cosmic-ray arrival time distribution can be used to create true and infinite random number sequences. However, despite many publications on this topic, a practical method for distribution of generated keys has not yet been presented. Addressing this discrepancy, COSMOCAT offers both key generation and distribution.

A novel technique for navigation and synchronization of distant clocks using the relativistic nature of cosmic-ray muons has already been proposed with muometric positioning^36^ and navigation^37^ and the Cosmic Time Synchronization by Tanaka.^38^ These techniques utilize the known speed of muons (near the speed of light in a vacuum) to measure the distance between vertically spaced detectors. This technique works inversely, such that if the distant clocks are already synchronized and if the sender knows the distance between the sender’s detector and the receiver’s detector, the sender will also know the exact cosmic-ray muon’s arrival time at the receiver. When this concept is applied to COSMOCAT, the cosmic key (digits of the muon’s arrival time) will be “delivered” (or decoded) from the sender to the receiver without physically exchanging information between senders and receivers. In this work, the first experimental results of cosmic key generation & distribution with COSMOCAT will be presented and its possible applications will be discussed.

## Results

### Principle of COSMOCAT

COSMOCAT utilizes the relativistic nature of cosmic-ray muons that are generated by the collision between primary particles and the atmospheric nuclei. When primary cosmic rays collide with atmospheric nuclei, high-energy interactions generate cascades of secondary particles. Muons are generated by high-energy meson (charged pions and kaons) decay processes within the atmosphere. These muons are mostly relativistic and penetrative. They have been used as probes for imaging gigantic objects such as volcanoes, ocean tides, and cultural heritage sites.^39^^,^^40^^,^^41^^,^^42^^,^^43^ Cosmic-ray muons lose only a small fraction of their energy, mostly by ionization, as they traverse through matter. The transverse momenta of the parent mesons generate the lateral spread of the muon component, but compared to the electron, the muon’s multiple scattering contribution is much smaller since it is suppressed by a factor of (*m*_e_/*m*_μ_)^2^. The direction of the primary particles is strongly affected as they travel through the random component of the galactic magnetic field. Consequently, by the time they arrive at the Earth’s atmosphere, their initial direction is completely lost. However, due to different atmospheric thicknesses and density gradients for different arrival angles of primary cosmic rays, the resultant horizontal muon flux is lower than the vertical flux. The COSMOCAT technique utilizes all of the muons arriving from the upper hemisphere. By utilizing these muons, we can establish a three-dimensional near-field COSMOCAT network that consists of three-dimensionally distributed COSMOCAT sensors for secure control and management of near-field wireless devices such as WPT and BAS.

In the COSMOCAT scheme, (1) if the sender knows the receiver will detect the same muons, (2) if the sender knows the distance between the sender and the receiver, and (3) if the time is accurately synchronized between the sender and the receiver, then the sender can predict the muon’s arrival time (*t*_RECEIVER_) at the receiver’s detector (D_RECEIVER_) such that:

(Equation 1) $$t_{RECEIVER}=t_{SEND}+T_{0}$$

where *t*_SENDER_ is the muon’s arrival time at the sender’s detector (D_SENDER_), and *T*_0_ is the time of flight (TOF) between the sender’s detector and the receiver’s detector. Since cosmic ray muons are relativistic, *T*_0_ is well approximated as:

(Equation 2) $$T_{0}=Dc^{-1}$$

where *D* is the distance between D_RECEIVER_ and D_SENDER_. The error (*δT*_0_) associated with *T*_0_ is defined as:

(Equation 3) $$\delta T_{0}=T_{0}\;\left(1-\beta \right)$$

where *β* is

(Equation 4) $$\beta =v/c={\left(1-\gamma^{-2}\;\right)}^{1/2}$$

and where *γ* is the Lorentz factor. For example, for a 3-GeV muon that is a typical energy of cosmic-ray muons, the muon’s speed is 99.95% of the speed of light in a vacuum. Therefore, the error associated with *T*_0_ would be 0.05% (e.g., for *D* = 3 m, the error associated with *T*_0_ would be 5 ps). In other words, these 2 participants know that they automatically both have the arrival time of the same particle without having to physically send the information to each other.

Therefore, resolving the condition (1) is the most essential issue for ensuring the success of COSMOCAT operation. The procedure to confirm that the sender and receiver both have detected the same muons (given that each particle detector is working correctly) would involve the development of a logical circuit with the following configuration and function. (A) The sender’s oscillator and the receiver’s oscillator are synchronized with each other and both output pulses with a period of *T*. (B) These pulses are split and fed into a counter unit to count the number of these pulses. (C) At the same time, these pulses are fed into a time to digital converter (TDC) as start signals. (D) Pulses corresponding to detection of the muons are discriminated and fed into the TDC as stop signals. By following the processes (A)–(D), lists of muon event information for each event including the detector identification numbers and the corresponding muon’s arrival times are generated at D_RECEIVER_ and D_SENDER_ (see Appendix). In units of ps, these muon’s arrival times (*t*) can be described by using a numerical sequence (*N*_*i*_) as follows.

(Equation 5) $$t=nT+\Delta t=\sum_{i}N_{i+12}\times 10^{i}\left[s\right]\text{,}$$

where *N*_*i*_ can have a value from 0 to 9, *n* is the number of counts in the counter unit, and Δ*t* is the time displacement between the start signal and stop signal which have been fed into the TDC. For COSMOCAT, *N*_*i*_ is used as an encryption key. If the frequencies of the oscillators at D_SENDER_ and D_RECEIVER_ (*f*_SENDER_ and *f*_RECEIVER_) are exactly the same, Equation 1 will follow the relationship:

(Equation 6) $$\left({nT}_{SENDER}+{\Delta t}_{SENDER}\right)-\left({nT}_{RECEIVER}+{\Delta t}_{RECEIVER}\right)={\Delta t}_{SENDER}-{\Delta t}_{RECEIVER}$$

where Δ*t*_SENDER_ and Δ*t*_RECEIVER_ are respectively the time displacements between the start signals from the sender’s oscillator (CLK_SENDER_) and the receiver’s oscillator (CLK_RECEIVER_) and stop signals from D_SENDER_ and D_RECEIVER_; hence, (Δ*t*_SENDER_- Δ*t*_RECEIVER_) is the muon’s TOF between D_SENDER_ and D_RECEIVER_. Therefore, if CLK_SENDER_ and CLK_RECEIVER_ are perfectly synchronized, the sender and the receiver will share almost the same *N*_*i*_ with an accuracy of *δT*_0_. However, in general, the key generated at the sender location does not match with the key generated at the receiver location. Since D_SENDER_ and D_RECEIVER_ do not have directional sensitivity, they detect open-sky charged particles in various angles independently; thus, the generated event lists contain random events which will be unique to either the D_SENDER_ or D_RECEIVER._ In open-sky environments, the fraction of the events used for keys to the entire events recorded in the list (*F*) is expressed as:

(Equation 7) $$F=\left[\int_{0}^{\varphi 0}\int_{0}^{\theta 0}I\left(\theta ,\varphi \right)d\theta d\varphi \right]{\left[\int_{0}^{\pi }\int_{0}^{\pi }I\left(\theta ,\varphi \right)d\theta d\varphi \right]}^{-1}$$

where *I* (*θ, φ*) is the muon spectrum, *θ* is the zenith angle, and *φ* is the azimuth angle. *θ*_0_ and *φ*_0_ are respectively the zenith angular region and the azimuth angular region formed by D_RECEIVER_ and D_SENDER_. The second term of Equation 7 indicates the open-sky muon flux that measures ∼10^2^ m^−2^s^−1^. Inside the building, the values of lower limits of integrals in Equation 7 are replaced with cutoff energies. The cutoff energies can be calculated by using the muon’s range-energy relationship.^44^

Due to these random events, the sender should send the encoded message repeatedly so that these random event errors can be eliminated and the receiver can successfully decode the message. In this case, there are only 2 options: either the receiver can decode the data or the receiver cannot decode them. When the receiver gets the encoded message, the receiver will try all the keys recorded in the event list for decoding as will be described in the following sections in more detail. In the current work, this matching key generation rate was ∼20 Hz out of total key generation rate of ∼10^2^ Hz; thus, this matching rate was roughly 20%. Therefore, the sender had to send the encoded message ∼5 times until the receiver could decode the message.

As can be seen in Equation 7, this matching rate is further reduced when the distance between D_SENDER_ and D_RECEIVER_ increases. The relationship between the key generation rate (*R*_K_), detector efficiency (*k*), and the matching rate (*R*_M_) is expressed as follows:

(Equation 8a) $$R_{K}\;∼1.5\;kIS$$

(Equation 8b) $$R_{M}∼\;k^{2}IS\Omega \;{R_{K}}^{-1}∼\;k^{2}{IS}^{2}D^{-2}\;{R_{K}}^{-1}$$

where *I* is the muon flux, *S* is the detector area, and *Ω* is the solid angle formed between D_SENDER_ and D_RECEIVER_. In a scenario with two detectors, the aforementioned logical circuits would be equipped inside both of the detectors to form COSMOCAT sensors. Additionally, temporal fluctuations in CLK_SENDER_ and CLK_RECEIVER_ and detectors need to be considered since they further degrade *R*_m_. Here, we label this electronics-associated temporal uncertainty as σ_*t*_. σ_*t*_ is generally much larger than *δT*_0_. The limitation of the key distribution rate due to this uncertainty will be discussed more as follows.

There are some limitations of the COSMOCAT technique that need to be addressed in order to make this idea more practical. Due to σ_*t*_, it is difficult for us to use the entire sequence of *N*_*i*_ as a key. Consequently, the number of digits we can use is up to σ_*t*_. For example, if σ_*t*_ is in an order of 10 ns, only *N*_*i ≥* 5_ will be guaranteed to be the same number for both the sender and the receiver.

**Figure 1:**
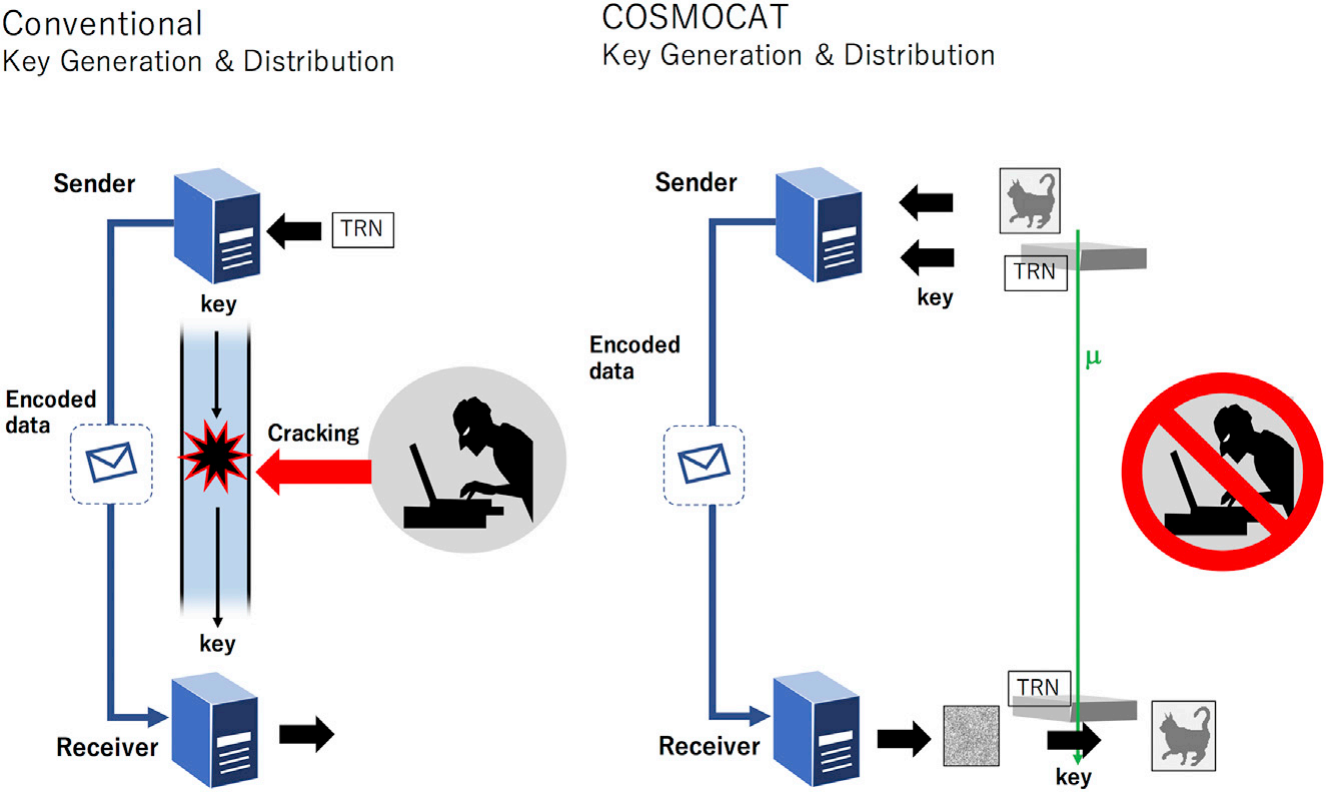
Conceptual comparison between the conventional key generation and distribution method and the COSMOCAT method The label TRN stands for the true random number generator. The symbol μ indicates a muon. With the conventional method, key distributors are independent from key generators. With COSMOCAT, muons generate keys and distribute them, allowing messages to be sent between them without physical key exchange.

Also, *N*_*i*_ will have to be converted to a combination of characters and numbers to strengthen the key. This conversion rule can be dynamically changed to further improve the key strength. In conclusion, sequences of true random numbers are continuously generated and renewed by cosmic-ray muons, and by using these as non-repeatable encoding keys (cosmic keys), highly secured communications will be possible since this cosmic key is distributed by muons from the sender to the receiver and thus from a distance it is impossible to crack this security. The concept of COSMOCAT is shown in Figure 1.

### COSMOCAT sensor

**Figure 2:**
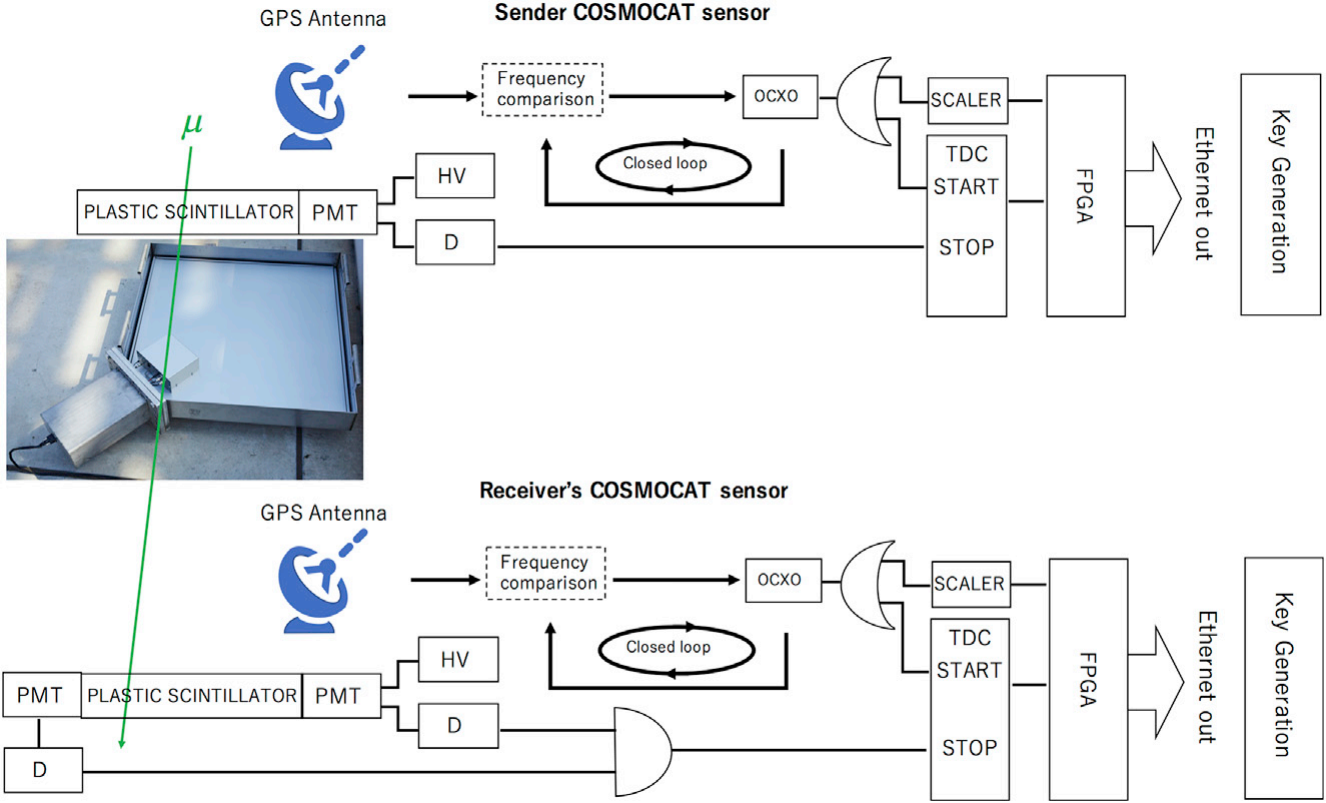
Block diagrams of the current COSMOCAT system The diagrams are shown for the sender’s COSMOCAT sensor (top panel) and the receiver’s COSMOCAT system (bottom panel). Labels PMT, HV, D, OCXO, TDC, and FPGA, respectively, indicate a photomultiplier tube, a high voltage power supply, a discriminator, an oven-controlled crystal oscillator, and a field-programmable gate array. A photograph shows one of the COSMOCAT sensors. The symbol μ indicates a muon.

The current COSMOCAT system consists of the sender’s COSMOCAT sensor and the receiver’s COSMOCAT sensor. Each sensor demonstrated in the current work consists of 1 × 1m^2^ square-shaped plastic scintillators with a thickness of 2 cm and photomultiplier tubes (PMT) (Hamamatsu R7724) that are connected to each corner of each detector via an acrylic light guide unit. A redundant PMT is attached to the receiver’s COSMOCAT sensor to reduce the accidental coincidence rate associated with the PMT’s dark current. Each COSMOCAT sensor has electronics that consist of high voltage supplies (HV), discriminators, the scaler electronics, and the TDC with a time resolution of 27 ps. Each COSMOCAT sensor is synchronized by a GPS/GNSS-disciplined oven-controlled crystal oscillator (OCXO) (GPS-DO) with a multiconstellation mode (GPS/GLONASS/Beidou/Galileo/QZSS) (Trimble Thunderbolt PTP GM200). HV is applied to PMTs, and discriminators are used for binarizing PMT outputs. The scaler electronics count the number of the pulses outputted from GPS-DO and the TDC measures the time displacements (Δ*t*) between the GPS-DO pulses and PMT pulses so that the event arrival times can be recorded as a function of the time counted from the start of the measurement. More specifically, the GMC pulses are fed into the scaler electronics to measure and output the value of *nT*, where *n* is the number of the GPS-DO pulses counted after the start of the measurement, and *T* is the period of the GPS-DO pulses. By transferring both of the time displacements (Δ*t*) and *nT* to a field-programmable gate array (FPGA) to merge these data, the time series of *nT* + Δ*t* is recorded in units of ps. The frequency of the OCXO has to be continuously corrected by the GPS signals. Single-count rates of the reference detectors and the receiver detector were found to be slightly larger than 10^2^ Hz. These processes are conducted in both of the sensors in parallel, and the time series of [*nT* + Δ*t*]_SENDER_ and [*nT* + Δ*t*]_RECEIVER_ is generated and respectively recorded in EED_SENDER_ and EED _RECEIVER_. A block diagram for this process is summarized in Figure 2.

### Randomness of the cosmic-ray muon arrival times

**Figure 3:**
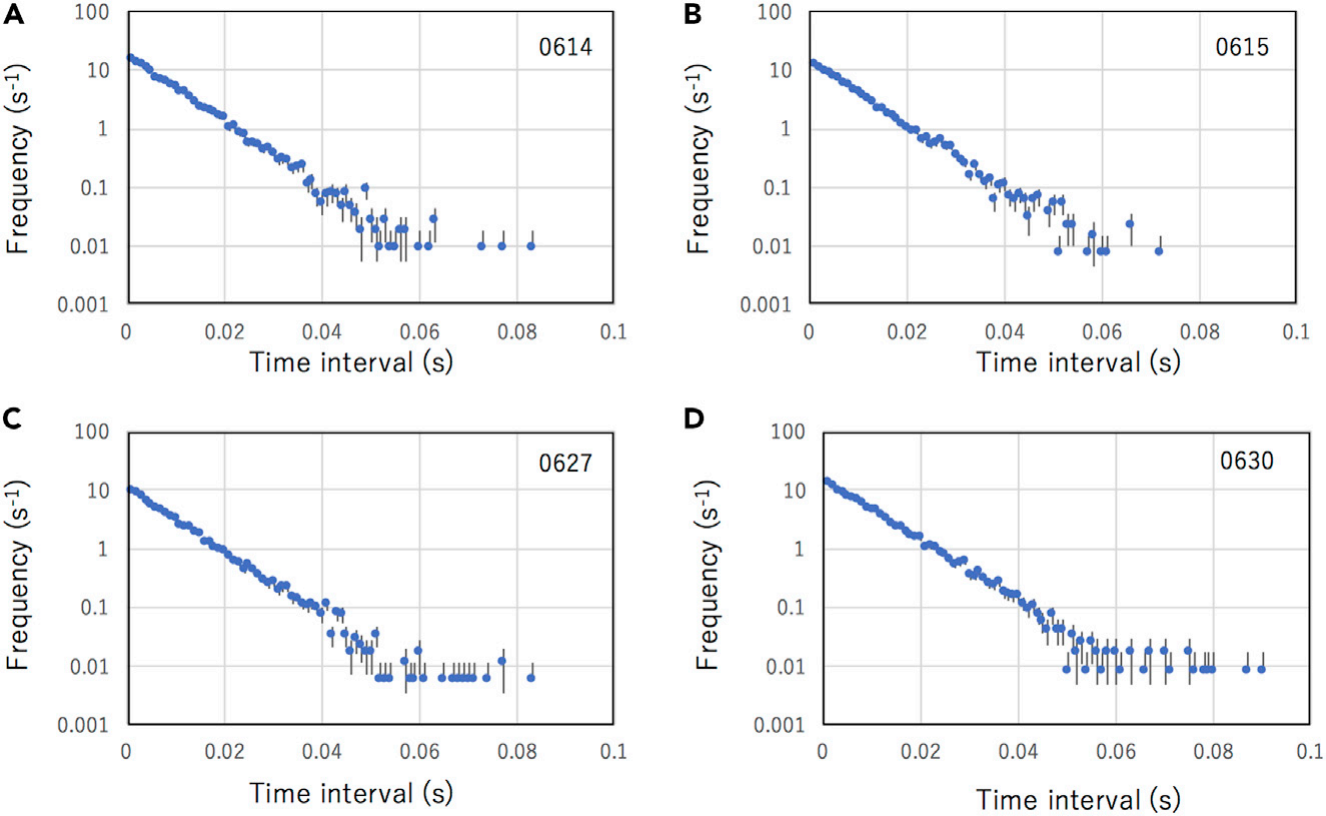
Time spectra Distribution of the time displacements between two consecutive cosmic events collected during each of the following days: June 14, 2022 (A), June 15, 2022 (B), June 27, 2022 (C), and June 30, 2022 (D). Error bars correspond to 1 standard deciation.

The quality of cosmic keys depends on randomness of the generated numbers. In the current work, the arrival time distributions of consecutive events have been measured and analyzed in terms of their random distribution function. The event data acquired with a COSMOCAT module were compared between four periods: (A) June 14, 2022, (B) June 15, 2022, (C) June 27, 2022, and (D) June 30, 2022. For the analysis of the time correlations, the time displacements between the consecutive events were investigated. The results are shown in Figures 3A–3D. The time distribution data were fitted to the gamma function (Poissonian process of order *M*) in the following way:

(Equation 9) $$G\left(t;\lambda ,M\right)=N\frac{{\lambda \left(\lambda t\right)}^{M-1}e^{-\lambda t}}{\left(M-1\right)!}\text{,}$$

where λ is the inverse of the mean value of the time displacement between two consecutive muons, *M* is the order of the distribution, and *N* is a normalization factor. For *M* = 1, Equation 2 is reduced to the following exponential function:

(Equation 10) $$G\left(t;\lambda ,1\right)=N\lambda e^{-\lambda t}.$$

As can be seen in Figure 3, the λ coefficient was almost the same (128.4–131.5 Hz) between these different dates. The fitted results of the time displacement distribution have a reasonable value, χ^2^/d.o.f. (0.98–0.99), indicating that the expected probability of observing *N* events in a time interval *t* is well described with a Poissonian process.

Additionally, in order to evaluate whether these numerical sequences are approximately the same as would be expected for a truly random sequence, NIST random generation tests^45^ were performed. Since the focus of the NIST test is the proportion of zeroes and ones for the entire sequence, these sequences were subtracted by the averaged number; ones and zeroes were respectively assigned to positive and negative numbers. According to the NIST Special Publication 800-22, if the computed P-value is >0.01, one can conclude that its associated sequence is approximately true-random. The current P-value calculated for *N*_*i*_ (15 digits) ranged from 0.18 to 0.37, and was sufficiently larger than the P-value suggested by NIST. Therefore, it was concluded that the numerical sequences generated with the current COSMOCAT modules were verified as true random numbers.

### Muon’s time of flight

**Figure 4:**
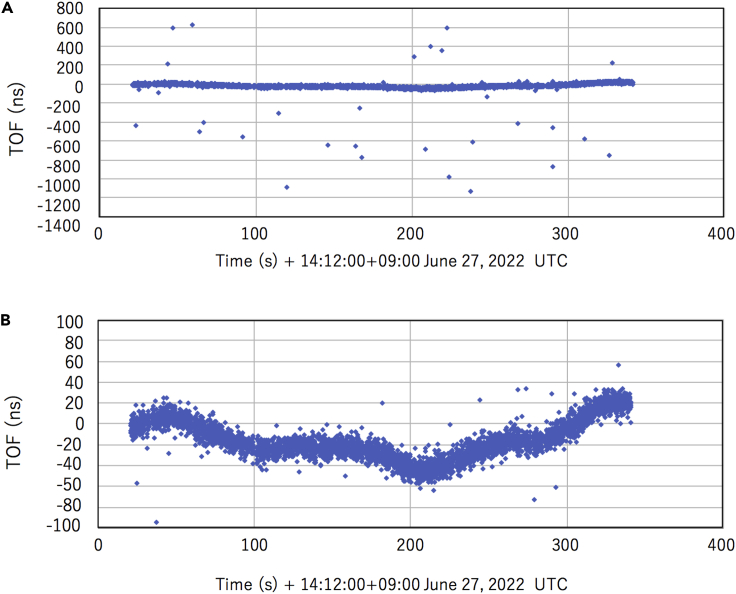
Time of flight (TOF) of muons measured between the periods 14:12:21 on June 27, 2022 and 14:17:41 on June 27, 2022 (A). A magnified view of the TOF is also shown for a time range between ±100 ns (B).

Figure 4 shows the TOF of muons measured between the periods 14:12:21 on June 27, 2022 and 14:17:41 on June 27, 2022. The number of matching keys measured in this period was 5,844 (∼20 Hz); this number is lower by a factor of ∼5 than the expected number. This event reduction comes from detection efficiency (<100%), accidental coincidence, and temporal fluctuations of GPS-DO. Since there was a relative frequency offset of ∼200 ns between GPS-DO1 and GPS-DO2, the TOF was normalized to the first sequence of TOF data we collected at 14:12:21. In the current work, the time window (*T*_W_) was set to be 2 μs for plotting dual coincidence events (Figure 4A). For *T*_W_, the accidental coincidence rate (*f*_ACCIDENT_) between D_RECEIVER_ and D_SENDER_ is defined as:

(Equation 11) $$f_{ACCIDENT}=2{R_{K}}^{2}T_{w}$$

Randomly distributed data points seen in this figure come from the accidental coincidence open-sky single events, with an average rate of ∼0.1 Hz (since *R*_K_^2^∼2 × 10^4^).

Figure 4B shows a magnified view of the TOF within a time range between ±100 ns. Since the expected TOF is ∼2 ns, which is negligible in comparison to the trend we can see in this figure, it was interpreted that this trend came from GPS-DO. As can be seen in this figure, there are 3 characteristics of GPS-DO that might affect the generated key quality: (A) jitters with an amplitude of ∼20 ns, (B) longer-period fluctuations with a cycle of a few hundred seconds, and (C) shorter period fluctuations with a cycle of ∼100 s. These fluctuations are a characteristic of the time-dependent relative frequency offset caused by the frequency drift of typical OCXO. These OCXO characteristics do not significantly affect the key generation rate (*R*_k_), but degrade the key matching rate (*R*_m_) by generating additional fluctuations in *N*_*i≤*5_.

### Cosmic keys

**Figure 5:**
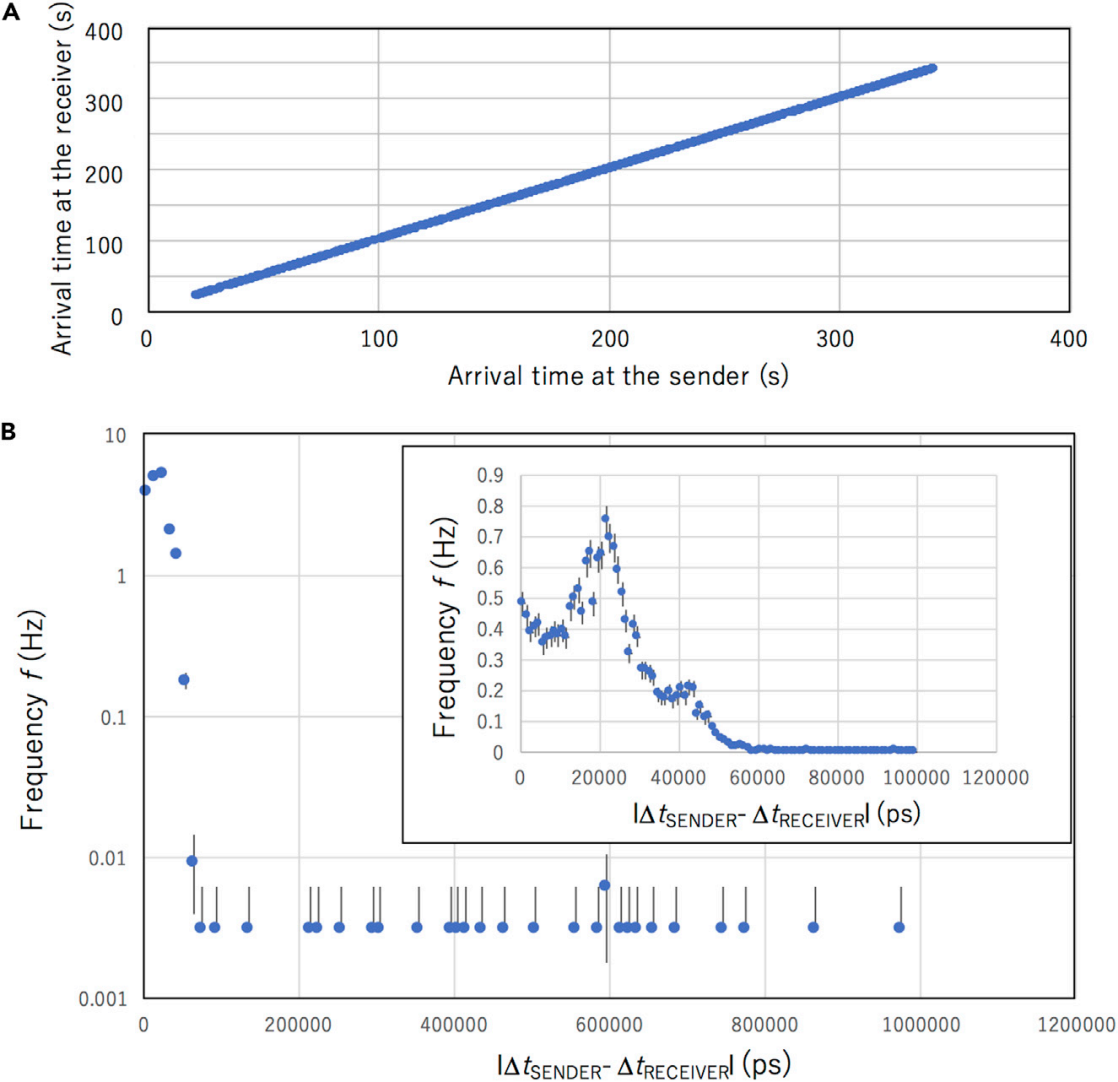
Cosmic key generation rate as a performance of the current COSMOCAT prototype The time series of the event occurrence recorded between 14:12:21 on June 27, 2022 and 14:17:41 on June 27, 2022 is shown (A). The distribution of the time displacements between the event occurrence recorded by the sender’s COSMOCAT sensor and the event occurrence recorded by the receiver’s COSMOCAT sensor |Δ*t*_RECEIVER_- Δ*t*_SENDER_| are shown (B). The inset depicts a magnified view of the same plot for a narrower time range (0–120 ns). Error bars correspond to 1 standard deciation.

The cosmic coding and decoding speed depends on *R*_K_ and *R*_M_. *R*_K_ was ∼10^2^ Hz in this work. Figure 5A shows the time series of the event occurrences (between 14:12:21 on June 27, 2022 and 14:17:41 on June 27, 2022) recorded by the sender’s COSMOCAT sensor as a function of the time of the event occurrence recorded by the receiver’s COSMOCAT sensor. Figure 5A indicates that overall, the generated receiver’s keys are close to the generated sender’s keys. Figure 5B shows more detailed time structure of the coincidence event. In this figure, the distribution of the time displacements between the event occurrence recorded by the sender’s COSMOCAT sensor and the event occurrence recorded by the receiver’s COSMOCAT sensor |Δ*t*_RECEIVER_- Δ*t*_SENDER_| are shown. Since the distance between the sender’s COSMOCAT sensor and the receiver’s COSMOCAT sensor was 70 cm, the muon’s TOF was expected to be 2 ns, but as can be seen in this figure, these time displacements were much larger than the expected value. The GPS-DO has a fluctuation width of ±50 ns peak to peak. Therefore, it was interpreted that the time structure shown in Figure 5B is due to these GPS-DO fluctuations. As a consequence, in this work, the time series of *N*_*i ≥* 5_ was used as cosmic keys. In the current experimental setup, *R*_M_ was calculated as the ratio between *R*_K_ and the rate of events tracked by D_RECEIVER_ and D_SENDER_ with a coincidence time window of 100 ns (*R*_*t*'*≤*100 ns_):

(Equation 12) $$R_{M}\;∼R_{t^{\prime }\leq 100\;ns\;}{R_{K}}^{-1}∼2\times 10^{-1}$$

where *t*' = |Δ*t*_RECEIVER_- Δ*t*_SENDER_|. *R*_*t*'*≤*100 ns_ was derived by integrating the time spectrum shown in Figure 5B over the time range between 0 and 100 ns such that:

(Equation 13) $$R_{M}=\int_{0}^{100\;ns}f\left(t^{\prime }\right)dt^{\prime }\approx 20\text{,}$$

where *f*(*t*') is the event frequency at *t*'.

### Performance examples

Cosmic keys are generated every time an event is detected at the sender’s detector. At the receiver’s detector, cosmic keys are also generated independently for decoding the message. These key generation rates were ∼10^2^ Hz for both sender and receiver with the current experimental setup. Since *R*_K_/*R*_M_ is ∼20%, four keys out of the five keys generated with the sender’s COSMOCAT sensor do not match *N*_*i*_ recorded in the receiver’s event list. Therefore, the sender should repeatedly send the message encoded with different keys, and the receiver has to try all of the keys listed in the receiver’s event list to find the matching key each and every time, and with the current setup, one out of the five encoded messages will be successfully decoded. These collating processes may seem laborious at first glance, but these processes can be automated, and the time required for these processes is negligible.

Therefore, the procedure the sender needs to follow is as follows:

(A) encode the message to be sent with the key (*N*_*i*_ (*t*_0_)) generated at *t* = *t*_0_,
(B) send the encoded message to the receiver,
(C) confirm whether the receiver could decode the message or not,
(D) if the receiver could decode the message, send the next message with *N*_*i*_ (*t*_1_).
(E) if the receiver could not decode the message, send the same message with *N*_*i*_ (*t*_1_).

The procedure the receiver needs to follow is as follows:

(F) decode the received message with the keys generated within the time range between *t* ± *T* s*.*
(G) send confirmation whether the receiver could decode the message to the sender.

The above processes can be all automated. The process (F) is the process to find the correct key to decode the message the sender sent. The number of keys (*n*_KEY_) the receiver needs to verify is:

(Equation 14) $$n_{KEY}={TR}_{K}$$

where, *T* = *CR*_M_. Although the average event rate tracked by D_RECEIVER_ and D_SENDER_ is *R*_M_, the key generation time interval is not always *R*_M_^−1^. Since this tracking rate is expected to follow the Poisson process, the tracking frequency *G* expressed as a function of the time interval (*t*):

(Equation 15) $$G\propto e^{-RMt}$$

Then the decoding failure rate (*R*_F_) is defined as:

(Equation 16) $$R_{F}=e^{-C}$$

**Figure 6:**
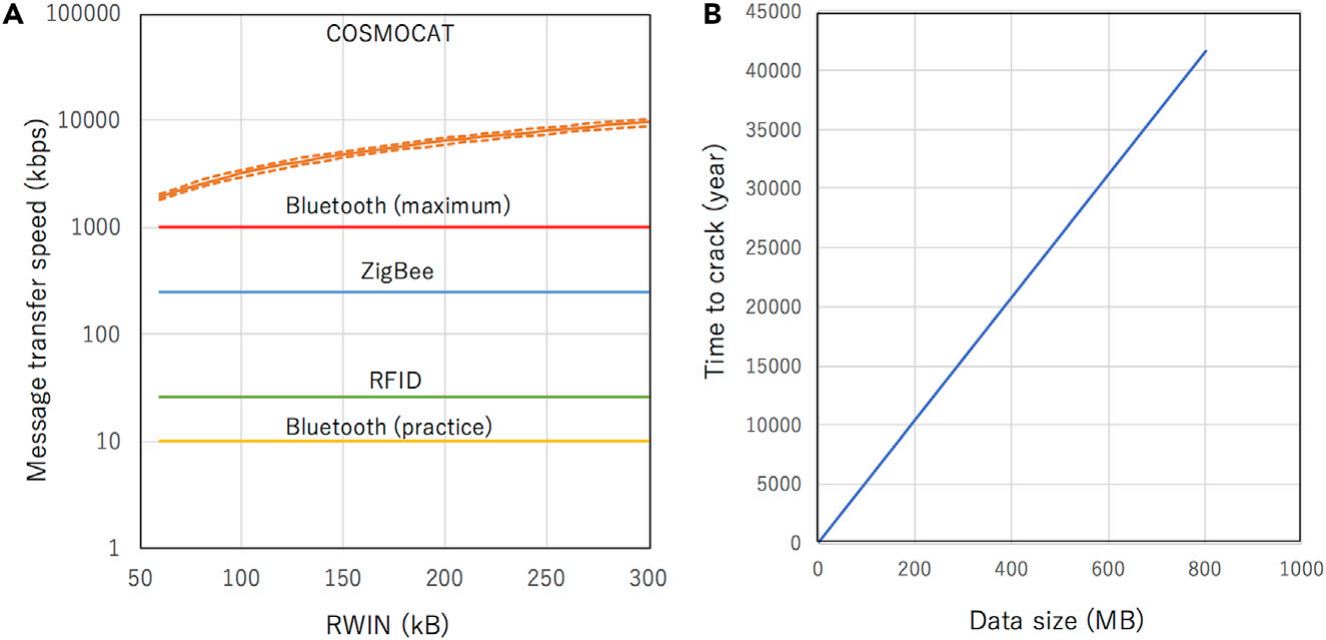
Comparison between the COSMOCAT message transfer speed and the speed of various NFC schemes at *R*_F_ = 0.7% (A). The COSMOCAT message transfer speed (orange curves) is plotted as a function of the size of RWIN. Orange dashed lines indicate the 1-σ upper and lower limit. The message transfer speed with other NFC schemes is plotted independent from the size of RWIN. The time required for eavesdroppers to crack the cosmic keys is also shown as a function of data size between the sender and the receiver (B). In this plot, an RWIN of 64 kb and a key size of 10 characters including numbers, alphabet letters, and symbols were assumed.

Therefore, for example, if *C* = 5, 99.3% of the receiver’s keys within this time range will match with the sender’s keys. Consequently, the processes (E) and (G) can be omitted. After all, adding the processes (E) and (G) to reduce *R*_F_ is equivalent to increasing the *C* value (or widening *T*). Here, we continue our discussion by assuming *C* = 5. In the current setup, *R*_M_^−1^ is ∼50 ms. Therefore, what *C* = 5 means that it takes 0.25 s to complete the process (F). Now, we estimate the attainable data rate with the following reasonable assumption. In the current analysis, the size of the message to be transferred was assumed to be equivalent to the fixed packet size (receive window size (RWIN): 65,535 bytes), and the time required for transferring this encoded message (round trip time: 15–25 ms) was neglected for simplicity. The time required for verifying the receiver’s event list and the decoded message sent from the sender to find the correct key is negligible (verifying 5,000 digits). If we encode every packet with COSMOCAT, the attainable data rate will be 2 Mbps at *R*_F_ = 0.7%. In this calculation, the processes (E) and (G) were omitted. This data rate is still much faster than the maximum speed of Bluetooth Low Energy (in practice 10 kbps), indicating that COSMOCAT offers an effective cosmic key generation and distribution for NFC. Figure 6A compares the COSMOCAT message transfer speed with the speed of various NFC schemes at *R*_F_ = 0.7%; this COSMOCAT message transfer speed is defined as a sum of the size of the message of which the sender can send with a moderate Wi-Fi network system per unit time and the size of the message of which the receiver can decode per unit time.

In practice, the numerical sequences *N*_*i*_ are converted to a combination of numbers, alphabet letters, and symbols to strengthen the keys. The number of digits that can be used for keys is 10 for *N*_*i ≥* 5_. Therefore, if only numbers are used for this key, it will take only 200 ms for eavesdroppers to crack this key, but if alphabets and symbols are added, it will take 5 months for eavesdroppers to crack this key with a brute force attack (identifying each digit of the key by enumerating all candidates) using a Gigabyte GeForce RTX 2080 Ti Turbo 11 GB Graphics Card.^46^ In the current scheme proposed here, since each packet is encoded, the time required for eavesdroppers to crack the message (*t*_CRACK_) increases in proportional to the number of packets used for sending the message, hence the total size of the message (*V*) such that:

(Equation 17a) V=(thenumberofpackets)xRWIN

(Equation 17b) $$t_{CRACK}=\left(the\;number\;of\;packets\right)x\;5\;months$$

This relationship is indicated in Figure 6B.

## Discussion

COSMOCAT technology has the potential to make a positive impact on all NFC and related secure information systems. The great advantage of COSMOCAT over other techniques is that (A) it includes practical methods for both cryptographic key generation and distribution, (B) it does not require any information traffic on regular internet or Wi-Fi for key distribution, and (C) since we do not emit signals actively, power consumption is low, hence providing the most robust and secure key transfer available. Information traffic requirements inevitably leave data vulnerable to hacking even with the quantum communication technique. For example, several quantum hacking attack strategies have been predicted and experimentally demonstrated in prior works.^20^^,^^22^^,^^47^^,^^48^^,^^49^ Also, it is practically difficult to design a perfect single-photon transmitter or receiver. Therefore, due to device imperfections, side-channel attacks could affect QKD systems.^20^ QKD is most vulnerable to an attack at the source side called a photon-number splitting (PNS) attack.^50^^,^^51^ The PNS attack occurs due to the use of a counterfeit weak coherent source which masquerades as the authentic single-photon source.^52^ Other powerful potential attacks are detector blinding attacks^53^ and time-shift attacks.^47^^,^^54^ For instance, as a “jamming” technique, the third party could send a bright light flash to the detector side powerful enough to disrupt the operation of the QKD detector. As can be seen in these examples, as long as there is information traffic, there is always a possibility this information traffic can in some way (known now or developed later) be jammed or hacked. The cryptographic community is in a race to deploy post-quantum cryptography robust methods and algorithms before quantum computers are spread throughout the society. COSMOCAT also assumes that the attackers have large quantum computers to break the present cryptographic systems based on, for example, the elliptic-curve cryptography approach. However, since COSMOCAT generates true random number keys, even with a presence of quantum computers, it is difficult to cryptanalyze within realistic time. Due to this aspect, we conclude that COSMOCAT offers one of the most robust NFC protections against cyberattacks, and is the next step for providing secure network solutions for vital information and operations. Regarding the power consumption of COSMOCAT, currently the most power consuming unit is OCXO (a few watts) since it requires heaters to keep the temperature of the oscillator. Other than this unit, FPGA (∼1 W) is power consuming. A PMT (∼200 mW), a TDC (∼70 mW), FPGA (∼1 W), and a GPS receiver (100 mW) are all small. In the future, OCXO can be replaced with an atomic (Cs) clock (∼10 mW).

**Figure 7:**
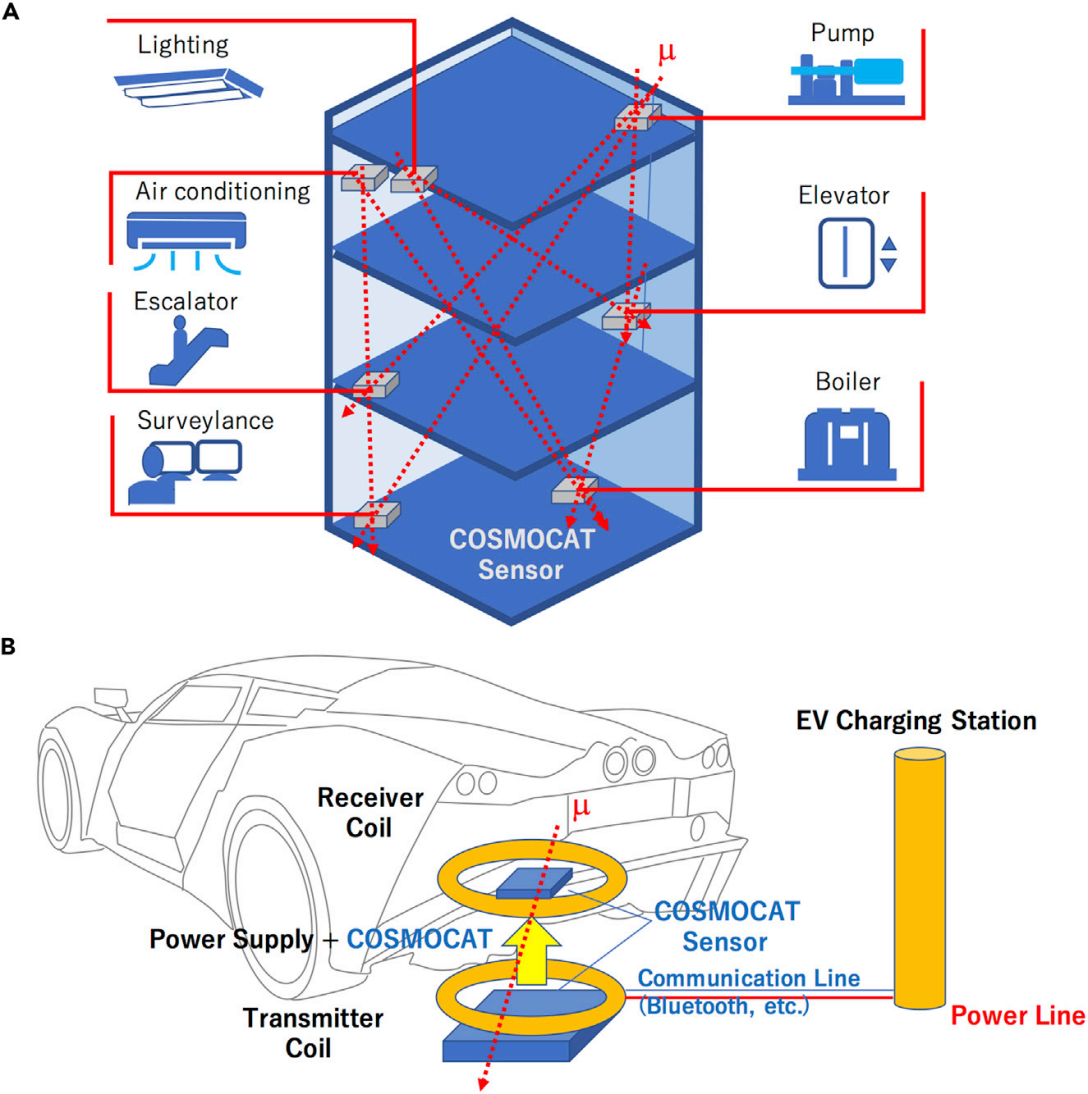
Examples of applications of COSMOCAT to NFC COSMOCAT sensors located near the devices for BAS generate cosmic keys to be shared between these devices (A). COSMOCAT sensors located near the receiver coil and the transmitter coil of the WPT station share the cosmic keys (B). Red dotted arrows indicate cosmic-ray muons.

Applications of COSMOCAT to NFC range from WPT to BAS. WPT is an emerging technology that is designed to be applicable to various transportation, logistics, consumer electronics, and biomedical healthcare requirements by enabling wireless charging between a wireless energy transmitter and a wireless energy receiver. Resonant inductive coupling is one of the major techniques currently envisioned for providing secure networking for WPT. However, this technique is associated with security vulnerabilities, for instance, energy stealing and/or power surge damaging energy receivers, since resonant inductive coupling is very sensitive to the existence of other energy receivers in the vicinity of the targeted energy receiver.^5^^,^^6^ COSMOCAT offers an unprecedented high security key sharing scheme that can be applied to resonant inductive coupling WPT systems without these risks. As part of BAS, functions such as lighting operation, air conditioning, escalators, surveillance, water circulation, elevators, water heating, etc. are all the targets of automation. These systems can all be all securely operated with a combination of COSMOCAT and various other NFC schemes. Examples of COSMOCAT applications are shown in Figure 7. In the current work, the size of the COSMOCAT sensors was 1 m.^2^ This size might be relatively large for practical implementation to the BAS devices. However, in many cases, we do not need 1–10 Mbps communication speed for controlling these devices. If the regular Bluetooth communication speed (10 kbps) suffices for operation, *ΩS* of the sensor can be compacted by an order of 2–3. Cosmically, keys can be used for end-to-end encryption of extension lines/secure telephone existing within a building.

Cosmically derived, randomly generated keys are difficult to steal. In order to steal cosmic keys, eavesdroppers would first have to install another COSMOCAT sensor between the legitimate, pre-installed COSMOCAT sensors. This action is generally practically impossible to implement because (A) eavesdroppers need to know the exact locations of the pre-installed COSMOCAT sensors, and (B) they need to install the hidden COSMOCAT sensor at a location with an identical solid angle to the one existing between the legitimate, pre-installed COSMOCAT sensors. For example, in Figure 7A, if D_RECEIVER_ and D_SENDER_ are located near the ceiling and the floor, since practically, eavesdroppers cannot locate their detector between these sensors, they would have to install the detector underneath the floor sensor to detect all the muons D_RECEIVER_ and D_SENDER_ track, and the size of their detector would be much larger than these sensors to cover the solid angle formed by D_RECEIVER_ and D_SENDER_ defined by Equations 8–2. Even if these actions are possible in principle, this would require many breaches of security in the physical location of the targeted victims, which is also usually practically impossible.

On the other hand, the weakness of the cosmic key generated by the current COSMOCAT scheme is that the first several digits are always repeatedly used for the keys. Even if these numerical sequences follow the true random numbers, there is a theorem that repeating usage of the same random number would cryptographically degrade the encoding strength. The actual strength of the cosmic keys generated by the current scheme must be further investigated in the future, but this is not the focus of the current paper. However, with the COSMOCAT method, in principle, keys are not exchanged physically and therefore cannot be leaked in the typical way.

In conclusion, the generation speed of the key that can be shared between the sender and the receiver is sufficiently fast that can support the data rate of the pre-existing NFC scheme. This indicates that COSMOCAT offers practical cosmic key generation and distribution to contribute to making the NFC system more secure in the future. The great advantage of COSMOCAT over other techniques is that (A) it includes practical methods for both cryptographic key generation and distribution, (B) it does not require any information traffic on regular internet or Wi-Fi for key distribution, and (C) since COSMOCAT doesn’t actively emit signals for key distribution actively, power consumption is low. COSMOCAT enables original, precious datasets to be safely encrypted with a true random number and these datasets can be securely transported to the receiver without being stolen or lost. As the modern infrastructure of society becomes more dependent on the safe storage and communication of valuable, secret, sensitive, and/or private datasets, we anticipate that the unique qualities of COSMOCAT have the potential to protect companies, individuals, and others from losing or compromising this valuable information, automation operations, and/or resource management. In this way, COSMOCAT may contribute positively to helping to defend valuable data from attacks in order to ensure that new technologies and ideas can expand and flourish.

### Limitations of the study

The distance between D_SENDER_ and D_RECEIVER_ must be shorter than ∼10 m for its practical use since the size of the detectors required for cosmic key distribution will be unrealistically large. In order to apply COSMOCAT to long-distant communication over 10 m, intermediate detectors to relay signals between D_SENDER_ and D_RECEIVER_ are recommended.

## STAR★Methods

### Key resources table

**Table:** 

REAGENT or RESOURCE	SOURCE	IDENTIFIER
**Other**		

PMT	Hamamatsu Photonics	R7724
Plastic scintillator	Eljen Technology	EJ-200
213">TDC	213">ScioSense	213">GPX
GPS-DO	Trimble	Thunderbolt GM200

### Resource availability

#### Lead contact

Further information and requests for resources and reagents should be directed to and will be fulfilled by the lead contact, Hiroyuki K.M. Tanaka (ht@eri.u-tokyo.ac.jp).

#### Materials availability

This study did not generate new unique reagents.

### Experimental model and subject details

Our study does not use experimental models typical in the life sciences.

### Method details

#### Material preparation

Our study does not materials typical in the life sciences.

#### Experimental apparatus

The current COSMOCAT system consists of the sender's COSMOCAT sensor and the receiver's COSMOCAT sensor. Each sensor demonstrated in the current work consists of 1x1m^2^ square-shaped plastic scintillators with a thickness of 2 cm and photomultiplier tubes (PMT) (Hamamatsu R7724) that are connected to each corner of each detector via an acrylic light guide unit. A redundant PMT is attached to the receiver's COSMOCAT sensor to reduce the accidental coincidence rate associated with the PMT’s dark current. Each COSMOCAT sensor has electronics that consist of high voltage supplies (HV), discriminators, the scaler electronics and the TDC with a time resolution of 27 ps.

#### Experimental procedure

The current COSMOCAT system consists of the sender's COSMOCAT sensor and the receiver's COSMOCAT sensor. Each sensor demonstrated in the current work consists of 1x1m^2^ square-shaped plastic scintillators with a thickness of 2 cm and photomultiplier tubes (PMT) (Hamamatsu R7724) that are connected to each corner of each detector via an acrylic light guide unit. A redundant PMT is attached to the receiver's COSMOCAT sensor to reduce the accidental coincidence rate associated with the PMT’s dark current. Each COSMOCAT sensor has electronics that consist of high voltage supplies (HV), discriminators, the scaler electronics and the TDC with a time resolution of 27 ps.

#### Calculation methods

See the main text.

### Quantification and statistical analysis

The fitted results of the time displacement distribution have a reasonable value, c^2^/d.o.f. (0.98-0.99), indicating that the expected probability of observing *N* events in a time interval *t* is well described with a Poissonian process.

### Additional resources

We have no relevant resources.

## Data Availability

The datasets used and/or analyzed during the current study are available from the corresponding author on reasonable request.

## Supplemental information

Data S1. D_Sender.csv The 10-second data collected at DSENDER. The first column indicates the detector identification number. The second column indicates the event identification number. The third column indicates the time of the event occurrence in units of ps.

Data S2. D_Receiver.csv The 10-second data collected at DRECEIVER. The first column indicates the detector identification number. The second column indicates the event identification number. The third column indicates the time of the event occurrence in units of ps.
